# Spirit of the British and American Periodicals &c.

**Published:** 1843-10-01

**Authors:** 


### Spirit of the British and American Periodicals.

---

### Kill or Cure.

The following Case, recently published by Mr. Hastings, will shew the fine effects of sudden cures by Hydropathy.

"*A Case of Gout, in which the Water-Cure was followed by Diseased Heart, Drcpsy, and Death. By* Charles Hastings, M.D. F.G.S.

"There exists no doubt in my mind that one great cause of the spurious celebrity of the Silesian peasant, Priessnitz, is that he has cunningly succeeded in turning the love of the marvellous, which is known to be a principle inherent in the human breast, to his own selfish ends. Every unprejudiced account which we receive from Graefenburgh confirms this opinion, by shewing that there is little faith to be placed in the reputed cures there performed.

"The cures said to be effected by the Hydropathists are as wonderful as those of Prince Hohenloe, and somewhat of the same faith which distinguished his disciples animates the hydro-maniacs.

"Dr. Pfeufer, directing physician of the Universal Hospital at Bamberg, thus describes the scene of operation of this princely priest, and the rush of the infatuated sufferers from disease to his presence:—'Let us then (he says) imagine thousands and thousands of human beings under such circumstances, to each of whom every day brings new sufferings, new despondence, and who have but one wish remaining—release by death from their nameless pangs. Electrified by the news that at Bamberg and Würzburg there is help and salvation for them through the grace of God, a new life glows within them, and already they feel the term of their misery. With the same longing as that of the leper for the pool of Bethesda, they hurry to the place where all grief vanishes, and where the stream of health and deliverance flows. In ever increasing expectation, and with breasts full of hope, wholly occupied in anticipating the enjoyment of re-established health, dead to every other thought, they approach the scene of healing grace. At every step are announced to them the great wonders that take place there every minute. Scarcely arrived at the place of deliverance, they throw themselves at the foot of the altar, confess their sins, and, reconciled and united with God, hasten to the chosen priest. All the streets they find filled; with great trouble and anxious palpitation they reach their desired residence. Some one carries on his back an old woman into the house; she has been, says report, from her childhood crooked, and unable to stand or walk. A few minutes pass away, the door of the Prince's dwelling opens, and the same woman walks without support out of the house, thanking and praising the Lord God. Every one presses around her; each one will with his own eyes, and from herself, learn what has happened to her. Man lives for the present time; of the past nobody has time to think; no one, therefore, concerns himself about the former situation of the person cured. Often, also, he who is not lucky enough to see or speak with her, still asserts, in order to please the crowd, and to appear of some consequence, that, with his own eyes and ears, he has convinced himself. For who is not gratified in being the bearer of wonderful news? And now, in an incredibly short time, the new miracle spreads through town and country; each narrator vies with the other. If the cured person limped at first, he is soon dead lame, and soon after sees him struggling with death; and the story becomes more interesting, and the narrator more consequential in proportion, as he is more skilful in tale-telling.'*

"It is not impossible that the system here described may take place in a greater or less degree in the hydropathic establishments in these kingdoms. Patients who have scarcely any real illness may be puffed off as having been released from grievous suffering; and if, perchance, any persons are just temporarily bettered by the aid of hope, imagination, and pure air, these cases may be magnified into important cures, and the future history of the invalid may be wholly lost sight of. Such, at least as far as I have had an opportunity of judging, has been the system pursued by the Malvern water-doctor. He has given to the world, through the medium of the press, as I am credibly informed, several important cures wrought at this salubrious village by the efficacy of the system of Priessnitz. One of these *cures* has happened to fall under my immediate observation, and, as I am desirous that *the truth, the whole truth, and nothing but the truth,* should be published concerning hydropathy, I will narrate the history of it.

"Mr. Probert, aged sixty-five, called upon me on the 11th of February, 1843. He told me that he had been for several years subject to attacks of gout, but in the intervals had been in tolerable health. He further stated, that he was attacked severely with gout the beginning of January, 1842, and remained ill with it for several months; but that in the month of July he was slowly recovering, and was enabled to get about a little. Some time in that month the water-doctor, who had lately arrived at Malvern, called upon him, and said that he could cure him in a fortnight, and, if he did not succeed, he would forfeit one hundred pounds. The poor fellow was delighted with the prospect of restoration to health, which had been denied to him for so many years, and, after a little consideration, consented to submit to the treatment. He was accordingly put under the feather bed and blankets for an hour or two, and when the perspiration came on he jumped into a cold shallow bath, and remained in it five minutes, and then walked about and drank water. This he did every day for six weeks. After this the gout left him, and he has not had it since; but his breath, which had been indifferent for several months previously, has ever since been much worse, and within the last two months has been so bad that he can scarcely move about. Within the last week his legs have swollen, and he is not able to lie down at night.

"The action of the heart is much diffused, and the impulse is great, with a *bruit.*

"I directed him to have a dozen leeches applied to the region of the heart, and afterwards a blister to the same part. He had also to take a pill of calomel, digitalis, and squill, three times a-day.

"On the 20th of March I again saw him, and the dropsical symptoms had disappeared, and he was rather better. As, however, the disease did not materially decline, he was, early in April, received into the Worcester Infirmary, and was then affected with general anasarca, urgent dyspnœa, and tumultuous action of the heart.

"He obtained some relief from active doses of elaterium, but did not on the whole derive much benefit, and, as it was evident that the mischief which had been produced in the chest could not be removed by medicines, he was discharged from the infirmary.

"After this he fell under the care of Mr. Addison, of Malvern, where he died in the month of June. Mr. Addison thus writes, in transmitting to me the report of the post-mortem examination:—

"'I visited Probert several times before his death, and found him suffering

* "Ed. Med. and Surg. Journ. Vol. XXV, p. 275."

greatly from general dropsy. His extremities were much swollen, and his respiration very difficult; he could not lie down, and during the last two or three days of his illness he was attacked with violent spasmodic paroxysms in the muscles of the arms, legs, and face, during which he cried out loudly from the acuteness of the pain. I do not remember to have ever witnessed so much suffering from spasms of this kind in cases of a similar description. I examined the body after death.

" ' The cellular membrane was everywhere distended with a yellow serous fluid.

" ' The lungs were crepitant, and the sections which I made, swam in water, still the tissue was at all points loaded with a serous fluid.

" ' The liver was not enlarged, but it was very pale and indurated. There was no great quantity of fluid in the abdomen, and the rest of the viscera in this region appeared healthy.

" ' The heart was nearly double the normal standard for persons of Probert's size, and the walls were in some degree hypertrophied. I examined this organ minutely, but I could not discover any ossific deposit in any portion of its structures, but there were several patches of coagulable lymph or fibrinous exudation, on the surface. The pericardium contained more than a pint and a half of serum.

" ' *June* 28*th*, 1843.' ' WILLIAM ADDISON.'

" *Remarks.*—The cure of gout by the water system is not novel. It has been repeatedly tried at different epochs of medical science, but has never, for any length of time, been enabled to maintain its credit as a *safe* mode of treatment. Within our own time, Dr. Kinglake attempted to bring this treatment forward as eminently successful. Serious and even fatal results were found occasionally to follow its adoption, and his plan never received the sanction of the profession. Gout is a disease of so migratory nature, and yet so essentially constitutional, that you can never say, when it disappears in one part of the system, whether it may not show itself in some other organ. It is well known that, in some broken-down constitutions, gout is never altogether absent from the system; and experience has taught us that in such cases the functions of the vital organs are better performed whilst the gouty action is showing itself in the extremities, and there is nothing in such a state of system which a prudent physician so much dreads as the sudden and entire disappearance of gout from the extremities. Probert's was just such a case. He had been an ailing man for years, and had been occasionally subject to attacks of gout in the limbs, which he considered were always followed by an improved state of general health. What does the water-doctor do in such a case? He comes and proffers his assistance, and actually forces his nostrum on the poor, unhappy sufferer! The gout, in consequence, disappears from the joints, and from that time forward his breath becomes affected. The gouty action is driven from the extremities, but it fixes on the tissue of the heart and pericardium, from whence it cannot afterwards be dislodged, and death at length releases him from suffering.

" Dr. James Johnson, in writing on this subject, justly observes, ' There are certain classes of maladies—for instance, gout, rheumatism, rheumatic gout, tic douloureux, &c.—which, though thoroughly constitutional, and whose *causes* have been years accumulating, are yet of a migratory nature, suddenly shifting their seat from a vital to an unimportant organ, and *vice versâ*, from a foot or a wrist to the stomach or heart. Now, it is an undoubted fact, that when a translation or metastasis of a chronic or subacute affection, as of gout or rheumatism, suddenly takes place from the exterior of the body, whether spontaneously or from the force of medicine, the malady takes up its seat in some internal organ; but as the internal organs—as the heart, liver, brain, &c.—are not naturally sensitive of pain, the metastasis is very often taken for a cure, and the malady preys for a long time on the vital part, without suspicion, until it reaches a certain height, when the disease reveals itself unequivocally, by pain and suffering, but is now totally beyond the power of art Nature will not be cozened by the ingenuity of man. For a long time she counteracts the deleterious effects of morbific causes, whether applied by ourselves, or unavoidably occurring, and guards vital organs by throwing the onus on external parts, as is familiarly exemplified by gout. But when we thwart these salutary efforts of Dame Nature, by violently repelling the pain, inflammation, stiffness, or swelling from the hands and feet, by cold applications, heroic doses of colchicum, &c., then we lay the foundation, directly or remotely, for serious or even fatal maladies of some of the internal viscera! In this drama HYDROPATHY is now playing an important part, and we are now almost in the daily habit of seeing the precious fruits of the 'water-cure' in the shapes of furuncles, carbuncles, dropsy, and hypertrophy of the heart,' &c.

"Further comment on the case of Probert seems unnecessary; but I may point out to the reader that this victim of the rash and absurd water-system had been known at Malvern as a gouty subject for several years, and there appeared, on the arrival of the water-doctor, no reason why he might not have continued in the same condition for as many more, if it had not, unfortunately for him, so turned out that the disciples of the Silesian peasant determined on making him one of their victims; but, instead of prolonging his days to upwards of one hundred years, which their system promised, they forced him to shuffle off his mortal coil in a few months, and made the grave his resting-place.

Worcester, July 22, 1843."

---

### AN ATTEMPT TO DETERMINE THE INFLUENCE OF THE SEASONS AND WEATHER ON SICKNESS AND MORTALITY. By WILLIAM AUGUSTUS GUY, M.B.*

This enquiry, Dr. Guy states, was suggested by a striking coincidence observed in the recently-published Report of the King's College for 1842, between the prevalence of sickness in the several seasons and the temperature.

"A desire to ascertain whether these were mere coincidences, or the general rule of sickness and mortality, prompted an examination of the records of sickness and mortality in past years; and the results of this examination are embodied in the present communication."

The enquiry consists of two parts. 1. As to the relation between the seasons and weather, and the sickness and mortality, during the year 1842; based upon the registrar-general's tables of mortality for the metropolis for that year, and the cases registered in the out-patients' books of the King's College Hospital for the same year. The standard of comparison employed is the meteorological table, deduced from observations made at the apartments of the Royal Society, and appended to the Registrar-general's report already mentioned. 2. A comparison of the results obtained for 1842 with those of former years.

We have not space enough to go through the valuable and interesting series of tables, &c. given by Dr. Guy; the following, however, are the conclusions which he draws.

"1. The amount of sickness in the central districts of London during the year 1842 varied directly as the temperature, being a maximum in August, the hottest month in the year, and a mininum in January, the coldest month.

2. The diseases which determined the order of sickness were febrile and

---

* From the Quarterly Journal of the Statistical Society.

catarrhal affections, the contagious exanthemata, and the disorders of the digestive organs; to which may be added the mixed group, consisting of gout, scrofula, &c.

3. The diseases of the organs of respiration followed the inverse order of those already mentioned, and were inversely as the temperature, being most numerous in the colder, and fewest in the hotter months.

4. The temperature did not appear to exercise a marked influence on the other classes of disease.*

5. The hygrometric state of the air appeared to have little effect on disease, and if it produced any effect, it was on the diseases of the organs of respiration, which were in excess during the months in which the quantity of moisture in the air was the greatest; but these were also the coldest months.

6. The mortality for the metropolis during the year 1842, was greatest in the first quarter, and least in the second, and was inversely as the sickness, except that the mortality of the third quarter exceeded that of the fourth.

7. The diseases which chiefly influenced the order of the quarters in respect of mortality, were those of the chest, to which may be added, as following the same order, the decay of nature in the aged.†

8. The order of the seasons in respect of sickness and mortality differs year by year, and does not admit of being reduced to any precise rule.

9. As a general rule, but one admitting of many exceptions, it may be stated, that the amount of sickness tends to vary directly, and the amount of mortality inversely as the temperature."

These results, though probably not very far from the truth, are based on too few facts to be certainly depended upon. In the course of the enquiry the following is the hypothesis which Dr. Guy was led to form.

The causes of sickness are two-fold, consisting of atmospheric changes which may be submitted to measurement, and of certain more subtle changes in the composition of the air, which at present can neither be analysed nor estimated. To the former class belong the temperature, moisture, and pressure of the air; to the latter those emanations from the earth or from human beings themselves, which give rise to the majority of epidemic, endemic, and contagious diseases. As the number of cases of sickness produced by these latter causes is generally considerable, the influence of the pressure, temperature, and hygrometric state of the air will not be observed in those years in which these causes are in operation; but in the absence of epidemics, the temperature will be found to be the most influential cause of sickness. When the temperature of the Summer is high, there will be such an amount of sickness in the Summer-months as to cause a large return of sickness for the entire year; so, on the other hand, a severe Winter will swell the total sickness of the year, by producing a great excess of affections of the organs of respiration. A Summer or Winter of unusual length, beginning early and ending late, will also cause an increase of sickness on the entire year, but the nature of the sickness will be different as the temperature is higher or lower than usual. The order of the seasons in respect of sickness will also be mainly determined by the degree in which the temperature of these seasons exceeds, or falls short of, the average temperature.

The mortality in like manner, in non-epidemic years, will be chiefly dependent upon the temperature, varying in the several seasons inversely as the tem-

---

* With the exception perhaps of those which form a measure of the activity of the sexual passion, which were in excess during the hottest months of the year—a fact which corresponds with, and corroborates our experience of the influence of the seasons on crimes against the person, &c.

† It is well known that the most common cause of death in the aged, is an affection of the lungs, called "bronchitis senilis."

perature, except in those years in which the Summer is unusually warm, when the mortality of the Summer may even exceed that of the Winter season. In other instances the mortality of the Summer months will rank next to that of the Winter or Autumn.

---

### ROYAL INSTITUTION—ELECTRICITY OF STEAM.

At the last evening meeting for the season, Professor Faraday communicated the result of his recent investigations connected with the electricity of steam. He stated, that attention was first directed to this subject in consequence of a mechanic, engaged in attending on a high-pressure steam-engine, having observed a spark to pass from the boiler to his hand while doing something to the valve. The man, with much alarm, reported that the boiler was full of fire; it was found, however, to be highly charged with electricity. Since then, several papers had been published on the subject by Mr. Armstrong, of Newcastle, in the *Philosophical Magazine*. It had been thought probable that it would afford some explanation of the origin of thunder-storms, the electricity being supposed to be produced, in both cases, by the evaporation of water. In confirmation of this view, the fact, which had been long observed, was adduced, that electricity was produced on the sudden evolution of steam caused by dropping water into a crucible heated nearly to redness. Mr. Faraday showed, however, that this effect resulted only under peculiar circumstances, and he said the experiments he was about to perform, entirely overthrew the supposed connexion between the electricity of steam and that of the atmosphere. His experiments were made with a small, high-pressure steam-engine, isolated from the ground by blocks of shellac. From this boiler the steam issued through a pipe, at the end of which was a spherical enlargement or globe, and beyond this a wooden tube with a small perforation. The pipe was furnished with a stop-cock between the globe and the boiler; and water was put into the globe up to the level of the pipe and wooden exit tube. On turning the stop-cock, the steam, passing over the surface of the water contained in the globe, escaped through the wooden tube, and the development of electricity was immediately manifested by a gold-leaf electroscope, connected with the boiler by means of a wire. The boiler was now found to be sufficiently charged with electricity to ignite hydrogen gas or gunpowder; and Mr. Faraday said, that Mr. Armstrong, in experimenting with a large and powerful boiler at a very high pressure, had obtained sparks through a space of twelve inches, the intensity being much greater than they could produce with the most powerful electrical machine belonging to that institution. The wooden exit-tube being now removed, and the steam allowed to escape immediately from the globe, through a large opening, no indication of electricity was afforded by the electroscope, although in other respects the arrangements remained the same as before; thus affording evidence that the phenomenon did not arise merely from the evaporation of water. The question then arose—did it depend upon the friction of the steam against the exit tube? This question was answered by the facts, that if the water was removed from the globe, and the whole course of the pipe allowed to become so hot that no water was deposited from condensation, the steam might be allowed to escape through the wooden exit-tube as before, without the development of any electricity. It thus became evident that neither the evaporation of water, nor the friction of steam, was the cause of the phenomenon in question; but that it arose from the friction of water carried with force through the wooden tube during the exit of the steam. It further appeared that the water, to produce the effect, must be in a state of purity; its contamination with a very small quantity of a salt, such as sulphate of soda, or chloride of sodium, entirely prevented the production of electricity. This was shown by dissolving a small fragment of sulphate of soda in the water contained in the globe, when the electroscope ceased to be affected; and even common river-water contained enough impurity to produce the same result.

Several experiments were performed to shew the kind of electricity produced under different circumstances. In operating as above described, the boiler was found to be charged with negative electricity; on introducing a few drops of oil of turpentine into the globe containing the water, positive electricity was obtained. In like manner, the steam, after issuing from the exit-tube, was found to be charged with electricity, and this was either positive, negative, or neutral, according to the distance from the extremity of the tube.—*Pharmaceutical Journal.*

---

### On some Preparations of Balsam of Copaiva. By Mr. Jacob Bell.*

Among several preparations of this medicine, Mr. Bell mentions the following.

When balsam of copaiva is boiled with liquor potassæ, the mixture separates into two portions, a white oily substance or emulsion, which floats on a yellowish clear liquid. After standing for a day or two, the upper stratum becomes quite clear, the potash being thrown down, and the residue consisting of essential oil. The clear liquid is a solution of the resin in combination with potash. When evaporated to dryness, it assumes the character of the soluble resin. Caustic soda may be substituted for potash.

This liquid is supposed to contain the most active and efficacious portion of the balsam. A small quantity of sweet spirit of nitre is added to it in order to increase the effect.

The following proportions have been found to answer very well. A mixture thus prepared is much less nauseous than the balsam; a dessert-spoonful of it may be taken twice or three times a day:—

Balsam of copaiva, two parts.
Liquor potassæ (or sodæ), three parts.
Distilled water, seven parts.

Boil for a quarter of an hour, then add—

Sweet spirits of nitre, one part.

Allow it to stand a few hours, then draw off the clear liquid by means of an orifice in the lower part of the vessel.

This, the alkaline solution of copaiva, Mr. Bell considers as in some respects the least objectionable of all the preparations which he has named. Being deprived of the essential oil which is generally considered to be the most irritating principle, it is mild in its action, and it is less nauseous than the other mixtures on account of the perfect union of the alkali with the resin.

---

### State of the Pupil in Injuries affecting the Brain.

In a clinical lecture on an interesting case of fractured skull with depression of bone, published in the Medical Gazette, Mr. Solly remarks:—

"In the examination of a case of this kind, then, it is extremely important for you to enter minutely into all those signs which indicate any injury to the brain. First, the mental condition—this was perfectly normal; he was quite

---

* Pharmaceutical Journal.

sensible and his manner natural. Next, the state of the pupils—the iris is placed before that expanded surface of the optic nerve, the retina, as an intelligent curtain to guard it from injury. The vital contrivances by which it acts, and by which its action is directed, are so beautifully perfect, that the extent of the opening of the curtain is indicative of the state of the nervous apparatus it is destined to protect, by preventing such an amount of light impinging upon it as would be liable to injure it. In disease of the globe of the eye, the dilated pupil indicates more or less pressure on the retina by some cause in the globe itself, such as a permanently turgid choroid, &c. But if with a healthy eye, but in connexion with a blow on the head, we find a dilated pupil, then we have a sign of some pressure on, or injury to, the nerve in its course within the skull, or the ganglia in which it terminates.

"The dilated pupil, then, indicates very serious injury to the optic nerve, or the nervous centres with which it is connected, though it may happen that, as in the case of very severe concussion, the injury is remediable. The contracted pupil, on the contrary, indicates an irritability of the nervous instruments, an undue excitement of their natural function, not an obliteration of it. You will sometimes see, in the case of injury of the brain, dilatation of one pupil and contraction of the other; where this is the case, you will find the most severe injury of the brain on the side opposite the dilated pupil."

---

### HOW TO MAKE LEECHES BITE.

The leech, which it is intended to apply, is to be thrown into a saucer containing fresh beer, and is to be left there till it begins to be quite lively. When it has moved about in the vessel for a few moments, it is to be quickly taken out and applied. This method will rarely disappoint expectation, and even dull leeches, and those which have been used not long before, will do their duty. It will be seen with astonishment how quickly they will bite.—*Med. Gazette, from Weitenweber's Beitr.*

---

### REMEDY FOR TOOTH-ACHE.

The *Nepeta cataria* of Linnæus is recommended by Dr. Guastammachia as a sovereign remedy for tooth-ache, whether it proceeds from catching cold or from caries. The leaves of the plant are to be placed between the affected tooth and the opposite one; this causes a copious flow of saliva, and in two or three minutes the more violent pains are relieved. If the patient cannot keep the leaves in contact with the diseased tooth, he must chew them, and the object is equally attained by the flow of saliva thus excited.—*Med. Gazette, from Filiatre Sebezio.*

---

### ON THE USE OF THE TINCTURE OF IODINE AS AN INJECTION, IN FISTULA ANI. BY CHARLES CLAY, M.D.*

After adverting to the beneficial results attending the iodine injection in cases of hydrocele, Dr. Clay mentions a case of fistula in ano, in which he was induced to try the effect of injecting the strong tincture of iodine through the canal of the fistula. The operation was followed by severe pain for a few minutes, with

---

* Medical Times.

a less degree of smarting, itching pain, for two or three hours after. On the second day the injections were repeated, the pain produced was equally severe with the first day. After this, the iodine was employed every other day for seven times, when the canal was found perfectly closed throughout, and its mouth entirely healed; no other treatment was adopted, except a little aperient medicine occasionally.

"To give iodine injections a fair chance of success," Dr. Clay observes, "they should be well thrown up by a good powerful syringe, (made of glass, as the iodine affects the metallic ones,) and the operator should be convinced that the fluid reaches the whole length of the canal, which in order to ascertain, he should, for the first and the second dressing, wrap a little lint or tow round a bougie, and pass it up the rectum before using the injection, when, if the fluid is conveyed properly, a portion will stain the lint on the bougie. In the case given above, the tincture could not be detected in the rectum after the second dressing. The result of this case was highly satisfactory, and gives room to hope that iodine injections will not only become highly valuable in promoting that peculiar inflammatory action, by which means the exhalents in the serous cavities are effectually obliterated and prevented from pouring out the secretions into those cavities, but also as equally valuable in closing up fistulæ, as in the case just recited. In fistulæ, however, the injection is required to be much stronger. I use the tincture full strength."

---

### Remedy for Cough.

The abrupt violation of the decencies of social existence is one of the most annoying consequences of coughing and sneezing. If you happen to have a catarrh, and are not endowed with sufficiently strong powers of mind to be able to resist a paroxysm of coughing, place the end of your fore-finger upon the end of your nose, if you have a nose. In the absence of this central organ, rub your eyes as if you had been magnetized, and look at your watch. If the fit of coughing does not pass off, you will at least pass for an accomplished gentleman.—*Diday.*

---

### Action of Weak Acids on Copper Vessels Plated by the Electrotype Process.

Mr. Warrington, in a late number of the *Chemical Gazette,* asserts that copper-vessels, such as saucepans, extract-pans, &c. silvered by the electrotype process, are acted upon by weak acids, as lemon-juice or vinegar, if allowed to remain in them for a short time. This, he says, must arise from the deposited silver being so porous as to allow the acids to permeate its substance, and the action is most likely assisted by the formation of a galvanic current.

---

### Observations and Researches upon a New Solvent for Stone in the Bladder. By Alexander Ure, M.D.*

Mr. Ure states that, in pursuing some enquiries relative to the treatment of certain forms of urinary disease, his attention was directed to the properties of car-

---

* Pharmaceutical Journal.

bonate of lithia, a substance of which no therapeutic application has been heretofore made. It occurs, nevertheless, as a constituent of various mineral waters, some of which have been found of service in certain unhealthy conditions of the urinary organs.

Carbonate of lithia dissolves in water at the ordinary temperature (60°) to the amount of one per cent. It possesses a faintly alkaline, by no means unpleasant taste. It has a remarkable affinity for uric acid; according to M. Lipowitz, one part of carbonate of lithia dissolved in water, and boiled along with an excess of uric acid, dissolves four parts of the latter, which are held in solution after cooling. Urate of lithia is, indeed, the most soluble salt which that acid forms.

In order to determine the solvent powers of carbonate of lithia, with reference to uric acid and its compounds, at the common temperature of the human body, Mr. Ure instituted several experiments.

On adding pure uric acid to a solution of one grain of carbonate of lithia in an ounce of water, at the temperature of 98°, the quantity taken up was found to be 2.3 grains. Hence, it appears that the solvent power of carbonate of lithia is more than double that of carbonate of soda; nearly double that of carbonate of potash or borax; and about eight times that of bi-carbonate of soda, which is the active ingredient of the Vichy water.

After keeping a human urinary calculus, consisting of uric acid with alternate layers of oxalate of lime, in a solution of four grains of carbonate of lithia in an ounce of water at the temperature of 98°, for five hours, it was found to have lost five grains of weight, being at the rate of one grain an hour. The calculus was deeply eroded in different parts, but the delicate laminæ of oxalate of lime remained intact, imparting to the surface the appearance of deep etching. The menstruum acquired a pale yellow tinge, and there fell down from it on cooling, a light flocculent deposit of urate of lithia, in which silky crystalline tufts could be distinguished by help of the microscope. It was still alkaline to litmus.

It deserves notice, that when fresh healthy urine is rendered alkaline by carbonate of lithia, no deposition ensues.

"A very large proportion," Mr. Ure observes, "of the stones which occur in the urinary bladder of man, are composed in whole or in part of uric acid. Of all the various menstrua hitherto recommended, none appears to promise more favourably than the carbonate of lithia, from the promptitude and energy with which in dilute solution it attacks calculi of this description. If by means of injection we can reduce a stone at the rate of a grain or more an hour, as the above experiment would lead us to anticipate, we shall not merely diminish the positive bulk of the calculus, but farther loosen its cohesion, disintegrate it, so to speak, causing it to be washed away in the stream of the urine. Cases may present themselves in which it may be expedient to conjoin the use of the lithontriptor; but only occasionally and at long intervals. It is the frequency of repetition which renders that instrument so hazardous,

"It may be presumed, moreover, that the plan of throwing in a weak solution of this kind, would generally exercise a beneficial influence in obviating irritation, by removing the sharp angular points and asperities of the broken fragments, where the practice of crushing is adopted."

Mr. Ure has been prevented from trying the carbonate of lithia, by its extreme scarcity.

It appears, however, that the mineral called *spodumene*, which is found at Killiney, near Dublin, contains, according to Stromeyer's analysis, 5.6 per cent. of lithia, so that the carbonate might be obtained without much difficulty, if a demand should arise for it.

### On the Treatment of Gonorrhœa, by Superficial Cauterization of the Urethra. By G. B. Childs, Esq.*

After some observations upon the prejudices which exist in the medical profession against "bold practitioners," Mr. Childs explains his mode of treating gonorrhœa at its commencement, when pain and inflammation are present, attended with discharge.

"Immediately a patient applies to me, I introduce an instrument, a modification of Lallemand's caustic-holder, smeared with oil, carrying it as far back the passage as from the symptoms may be deemed expedient. The caustic being exposed by pressing the stilet forward, the button at its extremity must be rapidly rotated between the thumb and fore-finger of the right hand, in order that no part of the mucous lining may be left intact, whilst the instrument is at the same time gradually withdrawn from the passage.

"In a few hours, a considerable degree of inflammation comes on, and in some instances slight bleedings, but these symptoms are but temporary, and subsiding leave the membrane almost free from discharge.

"In most cases of gonorrhœa the inflammation does not extend beyond three or four inches from the orifice of the urethra; this was called by Mr. Hunter its specific extent; further back than this, therefore, the instrument need not generally be passed.

"One application of the caustic I have sometimes found to destroy the virulence of the disease; but when, after the irritation attending the first application has subsided, any discharge remains, we should again resort to its employment. Whilst pursuing this treatment internal revulsives are to be administered in the form of copaiba and cubebs combined, and the penis is to be enveloped in a cold saturnine lotion.

"With such means as these we shall rarely fail to check the disease in a few days; an assertion I could easily corroborate by the recital of cases, did I feel it might be requisite so to do."

Mr. Childs disposes summarily of any objection to this plan on the grounds of the chance of stricture or epididymitis ensuing, by asserting that the chances of these affections are considerably less the earlier we destroy the specific inflammation attending gonorrhœa.

"The first and most important indication in the treatment of gonorrhœal urethritis is to make such an impression on the inflamed vessels as shall change the original character of the disease, and substitute, in its stead, simple common inflammation of a sufficient extent to overcome the diseased action; and I feel assured, nothing can so effectually induce this as superficial cauterisation."

---

### Evil Consequences of the too early Application of the Starched Bandage in a Case of Simple Fracture of the Fore-arm. By D. M'Cash, Esq.†

Some time since, Mr. M'Cash was called upon to visit a boy twelve years of age, who had just sustained a fracture of both bones of the fore-arm, about mid-way between the wrist and elbow. Unfortunately, the author was fresh from reading some glowing accounts by Velpeau of the successful application of *la bandage immobile,* in cases of simple fracture newly contracted, and, pleased with the nature of the details, resolved on giving it a fair trial in the first suitable

---

* Medical Gazette, July 28. † Medical Gazette, July 21.

case that should present itself. Accordingly the arm was at once encased within the folds of the starched bandage. All appeared to go on well, but the following proved to be the state of the parts some time after the apparatus had been removed.

"The fracture was firmly united, and the callus was nearly all absorbed. The fleshy part of the arm unusually round and firm, conveying the impression, when handled, as if the muscles were all matted together. Flexion and extension, save to a very limited degree, quite destroyed. The wrist drooped over the end of the splint, and was beyond the control of the patient. The fingers were rigidly hooked upon the palm of the hand, and likewise defied all attempts on the part of the patient to make them straight. Sensation undiminished. Arm a little emaciated, and rendered totally useless as an organ of prehension. The tendons of the flexor muscles were prominent at the wrist, and resembled contracted cords passing on the hands."

It would appear that there were all the usual consequences of acute inflammation terminating short of the formation of pus; thickening and condensation of all the tissues; a copious deposition of organised lymph around and into the substance of the muscles; morbid adhesions of one part to another, with all the other changes due to the same cause, to which alone the loss of motion of the limb was attributable.

Mr. M'Cash concludes justly, that "no prudent surgeon should overlook the guidance of his own judgment in the promulgated advices of authors and inventors, however authoritative they may seem, as to resort to such constricting measures, in the treatment of fractures, as the starched bandages before the inflammatory period which succeeds every accident has gone over, and the parts have subsided into their due medium of action. Such is the proper period for deriving its full advantage, but to apply it before this time is to impose a control upon nature which she will not suffer, and consequences most prejudicial to the reputation of the surgeon are almost certain to ensue."

Three years have now elapsed since the occurrence of the accident, and the arm remains in the same unserviceable condition.

---

### ON THE EFFECTS OF THE TER-CHLORIDE OF CARBON IN CANCER AND OTHER DISEASES. BY E. W. TUSON, Esq.*

Mr. Tuson states that he has employed the ter-chloride of carbon for some time as a medicinal remedy, at the Middlesex Hospital. It was ordered at first as a local application for a patient suffering under cancer of the breast; one drachm of the ter-chloride was mixed with a pint of water; linen rags, moistened in this lotion, were applied to the tumor. The effect was immediate relief from pain, and the fetor from the discharge was completely destroyed, the patient being comparatively comfortable. The medicine was then given internally, one drop in water three times a day. This dose was increased to two and then to three drops; the effect was sedative, producing sleep for twenty-four hours. The cancer soon afterwards sloughed, and considerable pieces came away; the surface left was irregular and excavated, having a healthy granulated appearance, with hardness, to a certain extent, round the surface.

The remedy was then applied to a large elevated cancer in the groin. It produced the same sedative effect, relieved the pain, produced sleep, and removed the fetor from the discharge. After the application had been used for some time, there arose an areolar inflammatory action around the tumor, of an erysipelatous

---

* Lancet, July 15.

character; this extended in the first case to about two inches, in the other to four or five inches, the skin around was red, of a deeper colour towards the disease, pitting under pressure, and leaving a white mark for a second. As the redness increased, large veins became visible, passing in a radiated and tortuous direction from the disease to the surrounding healthy parts; the slough next took place, when all this appearance gradually diminished, and the surrounding parts became again in a healthy state. The same application was employed during these changes.

Mr. Tuson is of opinion that this chemical preparation has some peculiar effect in cancerous patients, and in some suffering from other diseases, acting as a powerful sedative; but when employed in some other cases it had no such sedative effect. The following are cases in which he has employed it advantageously.

In *gangrena senilis,* its local application was of great service; its antiseptic property was here very remarkable, for where it was used by a patient, the fetor being so great as almost to prevent people from approaching the bed, the smell was soon much diminished. It also relieved pain and produced sleep after opium had failed.

In *sloughing ulcers,* its local application is said to prevent the slough extending, and greatly assist in its removal, rendering the parts beneath of a healthy character, and, in cases accompanied with much pain, giving immediate relief.

In *uterine affections, carcinoma, scirrhus, ulcerated surfaces, with profuse discharge,* its use as an injection produced great benefit; where all other medicines had failed, this frequently proved a valuable remedy.

In *neuralgic affections,* its local and internal exhibition proved often of service. In some cases of severe sickness, dependent upon nervous irritability, after the usual remedies had failed, this occasionally gave relief. "It allays nervous irritability, removes anxiety of mind, invigorates and raises the spirits, and where patients have one day been in a state of complete misery, they have on the following one become happy and joyful from its effects."

The ter-chloride of carbon is a clear transparent fluid, smelling strongly of chlorine, as its name implies; it consists of three parts of chlorine and one of carbon; the dose from one to four drops in water two or three times a day; one to two drachms in a pint of water, as an injection or lotion.

---

### Emplastrum Cerati Saponis.

The following are the directions given for the preparation of this plaster in the *Pharmaceutical Journal.*

The addition of one part of ceratum saponis to two parts of emplastrum plumbi, forms a plaster which, on an emergency, might be used when emplastrum cerati saponis is ordered in a prescription, the article in question not being a preparation of the Pharmacopœia. But the most legitimate mode of reducing the cerate to the form of a plaster, is to continue the evaporation until all the vinegar is expelled. This method is adopted at the Army Laboratory, where the plaster is kept, not only spread as usual, but also in rolls like other plasters.

The following is the formula used at the Army Laboratory :—

| | |
|---|---|
| Common vinegar (No. 24) .. .. | 8 gallons, old measure. |
| White Castile soap .. .. .. .. | 16 lbs. |
| Yellow wax .. .. .. .. .. | 20 lbs. |
| Olive oil .. .. .. .. .. .. | 32 lbs. |
| Litharge .. .. .. .. .. .. | 32 lbs. |

Boil the litharge with the vinegar almost to dryness; remove from the fire and add the soap, previously scraped or cut; replace on the fire, taking care to avoid empyreuma: then add the wax and oil, melted and strained, and continue the evaporation, stirring constantly until the whole of the vinegar is expelled. The time required for completing the above quantity is three or four days.

The original recipe directed eight gallons of the residuum of distilled vinegar to be used instead of twenty-four gallons of common vinegar. The plaster thus prepared has a dark-brown colour; but when the vinegar is used, and the plaster has been carefully made, its colour is not much darker than that of adhesive plaster. The emplastrum cerati saponis extensum, as usually sold, is coloured artificially with burnt sugar.

---

### ON THE SENSIBILITY OF THE GLOTTIS AFTER THE PERFORMANCE OF TRACHEOTOMY. By JOHN E. ERICHSEN, Esq.*

In the discussion which took place some time since at the Royal Medical and Chirurgical Society, on the highly interesting paper of Sir Benjamin Brodie, some discrepancy of opinion appeared to exist as to whether the sensibility of the glottis to the presence of a foreign body is diminished by an opening previously made in the trachea. Mr. Erichsen determined in consequence to institute some experiments on one of the lower animals, in order to arrive at some satisfactory conclusion on the subject.

The result of these experiments appears to be as follows:—In all the cases, whether the trachea had been previously opened or not, on irritating the larynx and glottis by means of a probe, attempts at coughing, violent spasmodic closure of the glottis, and convulsive action of the muscles of respiration and of the neck, were induced. But in those cases in which no opening into the trachea had been made, or, at least only one of such size as but just to admit the probe for the purpose of irritating the larynx, in addition to the effects above-mentioned, symptoms of incipient asphyxia speedily came on, produced by the spasmodic closure of the glottis arresting the respiratory changes, and thus occasioning the symptoms and sensations of impending suffocation.

Mr. Erichsen concludes, from these observations, that the existence of an opening in the trachea, sufficiently free to allow of respiration being carried on through it, or, indeed, complete division of that tube, does not materially, if at all, diminish the sensibility and contractility of the glottis. When a foreign body, therefore, accidentally introduced into the air-passages, escapes through the glottis without exciting spasmodic contraction of its muscles, or reflex movements in those of respiration generally, after an opening has been made in the trachea, it probably does so in the same accidental way that it entered; the sensitive parts through which it passes being taken as it were by surprise, whilst the attention of the patient is directed to the artificial opening, or to the circumstances in which he is placed. It would probably be as difficult for a patient (whether his trachea were opened or not) to expel a foreign body through his glottis, if his attention were fixed upon that part whilst he made the attempt, as it would be for him *voluntarily* to introduce it into the air-passages through the same aperture. There is, however, this most important difference between the presence of a foreign body in the larynx, or at the glottis, before and after tracheotomy has been performed, that, although the sensations of local irritation, and the reflex movements consequent upon them, may in both instances be the same, yet danger from asphyxia can necessarily only occur in those cases in which the glottis is the sole aperture, through which the respiration can be carried on.

---

* Medical Gazette, July 14.

Hence it follows that, in these cases, tracheotomy ought to be performed, not in order to facilitate the passage of the foreign body through the glottis, by diminishing the sensibility of that part, but in order to allow it a free exit through the artificial opening, which is not endowed with so extraordinary a degree of sensibility.

Another point is, whether in those cases in which some heavy foreign body has found its way into the air-passages, it would not be advisable, after the performance of tracheotomy, and before putting the patient into a prone position, to introduce some instrument into the opening in the trachea, so as to prevent the foreign body from being thrown, during the change of position, into the larynx or against the glottis, and thus occasioning much distress to the patient and embarrassment to the operator.

As the sensibility of the parts is not materially lessened after the trachea has been opened, Mr. Erichsen thinks this to be a sound piece of advice, and has accordingly invented an instrument for this purpose, the use of which might be attended with some advantages.

---

### On the Presence of Spermatozoa in the Fluid of common Hydrocele. By E. A. Lloyd, Esq.

At a late meeting of the Royal Medical and Chirurgical Society, it was announced that Mr. Lloyd had found, by the aid of the microscope, numerous spermatozoa in the fluid drawn off by tapping in two cases of common hydrocele. The first case occurred in the early part of last Winter. About three months afterwards the second case occurred. The patient was 63 years of age, and had been operated on for hydrocele about fifteen times. Sixteen ounces of a highly albuminous greenish-yellow fluid were drawn off. The author counted forty of these animalcules in one drop of this fluid; some of them were observed to retain their power of motion for three hours after the fluid had been evacuated. Blood-globules, transparent cysts, and small granular bodies, with portions of epithelium, or what much resembled it, were likewise found in the fluid.

At a subsequent meeting of the Society Mr. Childs stated that he had met with a third case of hydrocele in the fluid of which an immense number of spermatozoa were present. The fluid was of paler colour than that of common hydrocele of the tunica vaginalis, and very much resembled water with which a very small quantity of milk had been mixed. When tested with nitric acid, and also with heat, the fluid was found to contain a considerable quantity of albumen. There was also much saline matter in it. When examined with the microscope from three to four hours after it was drawn off, there were seen spermatozoa in a living state, and also an immense number that were dead: moreover the fluid contained a few blood-discs, transparent cysts, granular bodies of different sizes, and epithelial scales. The testis and its appendages were healthy. From the situation and form of the tumor it appeared that the liquid was contained in the tunica vaginalis.

Mr. Childs had examined the fluid of many hydroceles, but had never met with any animalcules except in these three cases.

---

### Dr. Watson on the Pathology and Treatment of Varices.*

Varices, which, according to the definition of Dr. Watson, are veins morbidly

---

* American Journal of Med. Sciences, Jan. 1843.

dilated, and usually elongated, convoluted and nodulated, may occur in almost any part of the body; the present paper, however, is confined to those in the vessels of the leg and thigh.

The changes produced in the vessels are, first, simple dilatation, which, though it may result from mere physical distention, soon leads to a proper interstitial development; the vessel then grows in length, and falls into folds and convolutions which occur most readily at those points where the least resistance is offered. The walls of the veins now yield irregularly to the pressure of the blood, constituting thrombi; these irregularities are at first easily effaced, but by degrees become fixed. The vessels contract adhesions to the surrounding tissues, which soon take on a diseased action, and become condensed into firm bands, uniting the different convolutions, preventing the vessel from exercising its proper elastic form for diminishing its calibre, and binding them into one mass in the form of a varicose tumor.

The next stage consists in the development of the elastic coat of the vessel; this, which is naturally extremely delicate, may become thicker and firmer than the corresponding coat in the largest arteries; it is then found to have a pale bluish, grayish or sometimes yellowish appearance; a cartilaginous hardness, and considerable contractile force. This hypertrophy may be either uniform, or partial, constituting nodules; these nodules may be numerous, and, when felt underneath the skin, giving the sensation of a hard knotted cord.

The inner coat appears to be the last to suffer. It becomes at last thrown into reticulated depressions, and afterwards into minute longitudinal folds, giving it, with the reddish tinge which it has now acquired, the appearance of delicate muscular tissue.

In consequence of these changes, the circulation becomes languid, the skin is engorged with venous blood, troublesome ulcerations break out, hæmorrhage not unfrequently takes place. The varices themselves are subject to attacks of erethism bordering on subacute inflammation. This leads to extension of the disease to other vessels, or induces ulcerations in their coats, giving rise to sudden hæmorrhages; or, finally, acute suppurative phlebitis may supervene spontaneously in the progress of varices, and lead to fatal consequences.

*Treatment.*—The treatment may be adopted with three views.

*First.*—To prevent engorgement of the limb; to keep the disease from increasing, or from exciting ulceration, or other concomitant affections; or to relieve these when existing. These indications are best effected by moderate compression, aided by elevation of the limb, simple dressings to the sores, &c.

*Second.*—To diminish the size of the vessels gradually and permanently. Varices may, in some cases, be gradually reduced by a change in the habits of life that gave rise to them. But they may be more rapidly reduced by special modes of applying pressure, by exciting inflammation in the tissues surrounding them, &c. Thus, Mr. Travers succeeded in obliterating a varicose cyst of the saphena, by means of adhesive plaster, applied in strips round the limb with as much tightness as could be borne. The vein inflamed, the cyst became a solid tumor which was at last perfectly obliterated. Or escharotics may be applied to the integuments over the affected parts; these, however, may give rise to diffuse phlebitis, to hæmorrhage or even to fatal consequences: and, if not carried so deeply as to excite adhesive inflammation in the vessel, they necessarily fail.

*Third.*—To obliterate the varices at once, and to force the blood into new channels. Aware of the ease with which the blood is returned by new channels after its proper veins have been obstructed, surgeons have long been in the habit of attempting the obliteration of these veins when diseased; and for this purpose have employed a great variety of measures.

*Laceration.*—This was in use among the early Romans. Caius Marius submitted to it on one leg, but very wisely declined having the operation performed on the other. It was effected by elevating the varicose veins and tearing them forcibly from their attachment.

*Cauterization.*—This also has long since been rejected.

*Excision.*—This was also practised by the ancients. Celsus was accustomed to expose the vein at several points, and then cut out portions of it.

*Erosion* has also been recommended and practised: but, in order that it may succeed it is necessary to apply the caustic deeply, and then it may give rise to abscesses along the course of the vessels, to alarming hæmorrhage, to diffuse phlebitis, and has ended fatally. Sir E. Home instituted the practice of employing ligatures; though occasionally successful, so many fatal consequences have ensued, that it has for the most part been abandoned. Soon after this, Sir Benjamin Brodie proposed the *subcutaneous incision*; this is recommended only when there are but few varices in the limb, and is performed on the branches, not on the main trunk of the saphena; even this has been followed by fatal inflammation. Of late years several distinct modes have been proposed of interrupting the circulation through the vessel by pins and ligatures, without exposing it. None of these, however, appear to be unattended with danger.

The operation which Dr. Watson usually performs in such cases as appear to him actually to demand such interference, is this; having rendered the vessel turgid, he makes an incision over the dilated vein, and raises it on a probe, other incisions are then made in proportion to the extent of the varices and the freedom of their anastomoses. Portions of the vein are then cut out in each of these situations, beginning with the lowest, the edges are brought together, and compression made by means of a roller. Afterwards the starched bandage is applied.

Several cases are related in which this operation was performed, but though usually successful, two instances are mentioned in which it proved fatal.

---

### Hydrocephalus, occurring at a particular Period of Life. By Henry Kennedy, M.B. &c. &.*

Dr. Kennedy presents us, in our Dublin contemporary, with an ample account of this affection. We confess that we have our doubts on its *hydrocephalic* nature. It appears to us to be rather, continued fever terminating in effusion within the cranium. We refer such of our readers as are curious with regard to particulars to the original paper. The following summary, drawn up by Dr. Kennedy, will give an idea of his views. He puts it in the shape of Propositions.

" 1. That an affection of the brain of the hydrocephalic character is not at all unfrequently met with between the ages of 12 and 25 years.

" 2. That it is more common in females than males, in the proportion of two to one.

" 3. That in the majority of cases it commences with symptoms of mild fever, which goes on without change for ten, twelve, and fourteen days.

" 4. That it sometimes begins by a distinct complaint of the head for some days, the patient still being able to go about.

" 5. That when the disease commences by fever, the first signs of anything going wrong take place very commonly at night.

" 6. That a marked increase in the degree of fever may then be observed.

" 7. That during the progress of the disease the pulse exhibits the characters of hydrocephalus, and to a marked degree.

---

* Dublin Journal, July, 1843.

"8. That alterations about the eye are often among the earliest symptoms pointing out that mischief is coming.

"9. That the pathology of the affection is confined in great part to the arachnoid at the base of the brain, with more or less effusion into the ventricles.

"10. That there are some grounds for supposing the inflammation to be of a specific character, probably strumous.

"11. That once the affection has fully declared itself, the treatment has yet to be determined.

"12. That local bleedings with mercury and blisters hold out the best prospect of success."

---

### Simple Mode of Treatment for Prolapsus Ani. By Dr. M'Cormac.*

We are afraid that the following news is too good to be true, viz. that prolapsus ani is to be cured by simply holding aside the skin round the anus, when the child goes to stool. Dr. M'Cormac, however, relates a case to that effect.

The subject of it had laboured under prolapsus from the age of one year till between five and six. The protrusion occurred at every stool, sometimes amounting to an inch or more, and had always to be reduced, a procedure attended with some difficulty, and more or less pain. The child had a relaxed aspect, was easily affected in her bowels, and evidently suffered in her general health. Dr. M'Cormac had tried everything that he could think of, short of an actual operation, painful to say the very least of it.

"Reflecting on the procedure in question, it occurred to me that the same result might in a measure, at least while the child was at stool, be secured by careful manual traction. I immediately stated my views to the intelligent mother; she entered into them at once, and promised, if possible, to carry them into effect. Accordingly when the child went next to stool, the skin exterior to the anus was drawn to one side by means of the fingers extended around. The little girl submitted to this with some reluctance, and complained that she could not evacuate her bowels. She was encouraged, however; a stool was obtained; from that day and date, now a month since, the bowel has not once descended. The stools, which previously were from two to four every day, have become much fewer, as well as of a more formed consistence and natural colour; while the child's health, spirits, and strength, are in other respects much ameliorated. There is now no prospect of the disease ever returning; the little girl requires comparatively little attendance; her mother, in fact, is only required to stand by, and in a short time, it is to be hoped, her onerous and anxious ministry will wholly cease."

The method is worth trying, but what with the attention it requires for a length of time, and what with the rather simple pretensions of the remedy itself, we feel some misgivings with respect to the result.

---

### On the Diagnosis of Valvular Disease of the Heart. By J. M. O'Ferrall, Esq.

Mr. O'Ferrall gives several cases and makes some very interesting remarks on the diagnosis of disease of the left auriculo-ventricular valve. Passing over the cases, we shall allude to Mr. O'Ferrall's views.

---

* Dublin Journal, July, 1843. † Ibid.

He observes, with justice, that when disease of the mitral valve proves fatal in its earlier or middle stages, the event is generally found to be owing, either to complication with renal, or some other chronic disease, or to one of the many accidents, in the lungs or pleuræ, to which this affection so often leads. Pulmonary apoplexy, pneumonia, bronchitis, or pleurisy, frequently interpose to prevent the completion of the morbid process. Now, in cases of this kind, witnessed by Mr. O'Ferrall, there was *a systolic bruit beneath the left mamma, persistent to the last, and looked on by him as characteristic.*

But, in some cases, this bruit disappears with the advance of the disease; and, in such, he has found the contraction of the opening so great, that *even the shortened valves were rendered capable of preventing a reflux,* and, consequently, performed their function once more. He asks, if it can be doubted that, should such cases be seen for the first time in this stage, by those who follow the text of Dr. Hope, a corresponding diagnosis would have been made, and the existence of valvular disease altogether denied.

"I am far," he adds, "from believing the rule to be, that in the advanced stage of disease of the left auriculo-ventricular opening, the phenomena of regurgitation shall cease. On the contrary, I have records of many cases, in which great contraction did not prevent the occurrence of a refluent current through the aperture. But in these cases, the inadequacy of the valve was still apparent, on inspection. I only maintain, that a valve, so shortened as to be incapable of closing the normal opening, may become adequate to its task, in consequence of progressive contraction, *combined with* a favourable adaptation of the aperture itself."

He subjoins the following propositions:—

"1st. That regurgitant disease of the mitral valve is attended by persistent murmur with the first sound.

"2nd. That the subsequent disappearance of this murmur does not lessen the value of the sign, nor contradict the diagnosis, at the time it was made.

"3rd. That the order of the phenomena here described in *combination* with the *general symptoms* of this disease, constitute a rational evidence of the supervention of contraction of the opening, to a degree proportioned to the previous shortening of the valve.

"4th. That uncomplicated obstruction of the aperture is not necessarily attended by a murmur.

"5th. That the general symptoms of disease of the mitral valve are not to be distinguished from those of *softening,* merely by the presence of murmur, as has been asserted by authors.

"6th. That the diagnosis can be made, only, by the observation, that a well-marked systolic murmur had *previously* existed, in *combination* with the *general symptoms* of the disease."

---

### On a peculiar Morbid Affection of the Stomach, characterized by Regurgitation of its Contents, without Nausea. By Sir Henry Marsh, Bart., M.R.I.A. &c.*

Sir Henry defines regurgitation to be that act whereby, without nausea, without convulsive effort, the contents of the stomach, gaseous, liquid, or solid, by a species of anti-peristaltic motion, are expelled. He remarks the utility of the function in the lower animals, particularly in the ruminants, as well as in the human subject, under particular circumstances. Thus the infant gets rid of the

---

* Dublin Journal, July, 1843.

superabundant quantity of milk—at all ages the gases of the stomach are so extricated—in pyrosis, fluid is thrown up.

In reference to *pyrosis,* we would make this observation. Without disputing the fact, that the fluid *may* come from the stomach, we are sure that, in many instances, it is the product of the *salivary glands.* We are ourselves prone to suffer from the affection, and have distinctly made out, in our own persons, that such is the fact. The peculiar sense of contraction and nausea at the pit of the stomach precedes a distinct and copious secretion from the salivary glands of the mouth. This fills with their secretion, and may be swallowed again and again until the flux subsides.

But even *rumination* may occur in men. Sir Henry relates two instances, the first, apparently, an unexceptionable one.

"I knew, many years ago, a remarkable example of rumination in a gentleman, who was a clerk in a bank; he enjoyed good health, lived at his desk, took but little exercise, and dined hurriedly, scarcely allowing himself time to masticate his food. Soon after dinner, portions of food, with little or no effort on his part, ascended into his mouth, were remasticated and again swallowed. In this manner, according to his own account, the whole of the food he had taken underwent this secondary process. It was a source of much enjoyment to him, and he prided himself upon the possession of this novel, but not very enviable, capability.

"I was called upon, not long since, to see a boy about fourteen years of age, and was informed by his mother that after every meal he regularly vomited his food, and that in consequence he had lost flesh, looked ill, and caused to his parents much uneasiness and anxiety. Upon examination, I found that there was no real derangement of the health, that his appetite was good, his bowels regular, and that the expression of his countenance did not indicate serious disease. By minute inquiry I ascertained that he did not vomit his food, but that it ascended from the stomach in successive portions, without nausea, without the least convulsive effort, and without any distress whatever to himself. I was present on one occasion whilst he was in the act of regurgitating his food; it was received into a basin; the morsels of meat successively returned had no acid taste, and did not appear to have been acted upon by the gastric juice. This occurred immediately after he had eaten plentifully and with an excellent appetite. The process of regurgitation was not preceded by any sense of distention or repletion; and whilst engaged in the act he talked and laughed as usual. He had acquired the habit, he could not tell how, since he had been sent to school, and it was quite obvious that he made use of it to induce his parents to remove him from school and to restore him to his horses, dogs, and gun. He was taken home, and there was a speedy termination of the supposed vomiting which had so much alarmed his parents."

Sir Henry observes, in continuation, that rejection of the food, without nausea, sometimes constitutes a distressing and intractable disease. It is seen in hysterical females. Of this he relates two instances. He then details the case of a girl, between 11 and 12 years of age, of stunted growth, in whom it followed diarrhœa and violent epileptic paroxysms. He adds:—"I have seen several other well-marked instances of the same disease in young children, but in every instance connected with, and resulting from, innate constitutional delicacy, and co-existing with various disturbances of the nervous system, and derangements of the digestive function. I have seen instances of it in conjunction with chorea. In many cases of hooping-cough, the partial or total evacuation of the stomach, at the close of the paroxysm, is the result, not of the act of vomiting, but of the act of regurgitation, and in such cases, so little is there of sickness or distress connected with the expulsion of the food from the stomach, that we often find the desire for food immediately to return, and children to eat greedily after having apparently vomited; but in many of those cases if the manner in which the food is expelled be closely examined, it will be found that it is accomplished without the effort and the sickness which characterize the act of vomiting. This distinction is of some practical importance, because it stands opposed to a method of treatment which is sometimes adopted,—that of treating hooping-cough principally by emetics. Doubtless, emetics are of use in the treatment of hooping-cough, and especially in such cases as are complicated with severe bronchitis. The frequent termination of the fit of coughing in the rejection of the contents of the stomach, has given rise to the notion, that the treatment by emetics is indicated; but if the manner in which the food is rejected be carefully examined, it will appear, that the practice of treating hooping-cough by exciting nausea cannot, in many instances, be fairly deduced from any natural indication."

In other instances, regurgitation forms one of the symptoms of obstinate and protracted dyspepsia. "In the same individual the food is sometimes vomited, and sometimes regurgitated; the contents of the stomach are sometimes rejected *en masse* without nausea or straining, and sometimes discharged morsel after morsel, till the whole of the contents of the stomach be disgorged. The food is in different individuals, and sometimes in the same individual, regurgitated at various stages of digestion, so that the ejected morsels present every variety of appearance and taste, from that which belongs to food just swallowed, until it be converted into perfect chyme, and mixed with the various morbid secretions which are found in a disordered stomach. Sometimes the act by which food and other matters in the stomach are propelled upwards, is accompanied with so much of nausea, and with so much of convulsive effort, as to give it rather the character of vomiting than of regurgitation, so that in fact it becomes not easy to distinguish the one act from the other. They run imperceptibly into each other, although in extreme cases, no two acts in nature can be more distinct."

Of this form of regurgitation, also, Sir Henry relates instances, and concludes the paper by some remarks upon the treatment.

For the hysterical and neuralgic form of regurgitation, the plan he has found answer best "consists of small blisters applied simultaneously to the pit of the stomach and to the spine; this, in some few instances, has been at once and permanently effectual; more generally, however, its salutary effects are only temporary; small detractions of blood by cupping in both situations have in some instances been equally successful. Benefit has been derived from small, and often-repeated doses of hydrocyanic acid, belladonna, morphine, stramonium, and other narcotics. Tonics, such as iron, bark, and bismuth, have also occasionally been found useful; but no remedy has been so often successful, in this form of the disease, as a total change of habits, change of air and scene, and travelling. In one case electricity was completely effectual in curing the complaint; it failed in others. In another instance, a sudden and powerful mental emotion totally and permanently removed the disease."

He has found it useful to advise the recumbent posture for an hour or more after each meal—to eat slowly, and to masticate the food well—to eat less than the appetite demands—to be moderate with fluids, and avoid distention of the stomach.

The affection has coexisted with tubercular disease of the lungs in not a few instances.

When combined with dyspepsia, "it is generally traceable to long-continued mental anxieties; to over thoughtful, studious, sedentary, and solitary habits; to the swallowing of food hastily without sufficient mastication and insalivation; to the utter neglect of the two most excellent promoters of healthy digestion—cheerful society, and full, free, enjoyable muscular exercise.

"In some cases it may be traced to excesses in sexual indulgences, and abuses of the sexual propensity." Every violation of the laws of Nature may give rise to it, and struma strongly predisposes to it.

An interesting communication.

### THE SALE OF ALUM TO BAKERS.

On examining the Act of Parliament, it appears that chemists and druggists are not liable to a fine for selling alum to bakers, although bakers are subject to penalties and imprisonment for using it in the manufacture of bread, or even having it on their premises. But, notwithstanding this law, baker's bread is seldom if ever free from alum. We have been informed by some of the trade that it is *absolutely necessary* in the quartern and half-quartern loaves, which would otherwise adhere together on being withdrawn from the oven, instead of separating with facility, which is generally the case. In cottage-loaves and other fancy bread, the bakers admit that alum is not so necessary, and its use, in these cases, is much less frequent.

Various contrivances are adopted to evade the law. Alum is called by bakers "*stuff*," or "*the doctor*." It is usually bought by the master, who deposits the proper quantity for each batch in some corner of the premises, where the foreman finds it at the proper time. In some houses the master is subject to a fine in case of his neglecting to provide "the doctor;" which fine is the perquisite of the journeyman. By these and other precautionary regulations, the inconvenience of detection is avoided; and although every person knows that alum is always used, no one is in possession of positive evidence of the fact, and all persons concerned keep their own counsel, being bound by that kind of "honour" which prevails "among thieves." The masters are interested in using "the doctor," because they can by this means improve the appearance of an inferior flour; and the men are equally interested in the matter, as the bread is made with less trouble. The parties aggrieved by this practice are those who consume the bread. Dr. Pereira, in his Treatise on Food and Diet, observes—

"Whatever doubts may be entertained as to the ill effects of alum on the healthy stomach, none can exist as to its injurious effects in cases of dyspepsia. Bread which contains alum is objectionable, not merely on account of this salt, but because it is generally made from inferior flour, which, when mixed with yeast and water, and formed into dough, quickly passes through the stage of vinous fermentation, and becomes acid."—*Pharmaceutical Journal.*

---

### MR. ROBERTSON ON EARLY MARRIAGES.

Mr. Robertson has been publishing in the *Medical Gazette* some observations upon early marriages in oriental countries as a proof of early puberty. The conclusions arrived at are these.

1st. That in England, Germany, and Protestant Europe in general, early marriage—that is, marriage about the age of puberty, would seem to be comparatively rare.

2nd. That early marriage prevails among the uncivilized tribes which wander within the arctic circle; as it likewise does in all cold countries without any exception, the inhabitants of which are in a state of ignorance and moral degradation.

3rd. That throughout European Russia, which is confessedly low in civilization, extremely premature marriage was the custom at no distant date.

4th. That, at the present day, in the most southerly countries of Europe, where the people are immersed in superstition and ignorance, marriage is early.

5th. That in Ireland, which, as to its moral condition, somewhat resembles the last-mentioned countries, the marriage union takes place among the Roman Catholic population at an age probably almost as early.

6th. That in England about two centuries ago, when debasing political and social circumstances combined to favour the practice, early marriages were general, at all events, in the upper ranks.

7th. That in all the countries to which reference has been made, juvenile marriage is invariably seen as an attendant on ignorance and moral debasement, and this without reference to *climate.*

8th. That consequently it is perhaps allowable to infer that early marriage in oriental countries, (which has generally, in the absence of all proof whatever, been ascribed to precocious puberty,) does solely depend on the same moral and political causes as elsewhere produce it; more especially as those very causes are well-known to exist, at present, in an aggravated degree, in all oriental and intertropical countries.

9th. That, instead of ascribing early marriage, so prevalent in our Asiatic dominions, to precocious puberty, (in the absence of all evidence whatever of the fact,) it would be desirable to try moral and legislative remedies, with a view to the removal of a practice so injurious; a practice which seems to be incompatible with social improvement.

---

### A Statement of the Mean Temperature of the Air, at Auckland.

A number of the Auckland Chronicle has been forwarded to us, containing some remarks on the climate of New Zealand.

It appears that, at Auckland, which is situated in lat. 36°.51. S., long. 174°, the mean temperature of the year is 58°. 26, of the Winter, 50°. 46, of the Spring, 56°. 18, of the Summer, 67°. of the Autumn, 59°. 03. In the warmest month, the mean temperature is 70°, in the coldest 49°.

From this it appears, says the writer, that the approximation of the temperature of Auckland to that of those towns in France most famed for their climate, is very striking, but with this superiority, that equality of temperature is much more favourable to Auckland than to the others: we may instance Montpelier, proverbial for its salubrity; there the difference between the mean temperature of the warmest and coldest month, is 35°, while at Auckland it is only 21°, making a difference of 15 degrees in favour of the latter—at Marseilles it is 30°, at Rome, 35°, even at Pau, in the south-west of France, latterly much frequented by invalids, from the supposed equality of its climate, the difference is still 32°, so that the climate of Auckland is milder in Winter and cooler in Summer, the variations of the temperature are less, and in these respects, it is much superior to all the above-named places.

Several fatal cases of consumption have doubtless occurred at New Zealand, but these it is said, were hopeless before arriving there, whilst some cases of pulmonary disease of a very aggravated character have been successfully treated, and succeeded by a more rapid convalescence than ever occurs in Great Britain. Epidemic diseases are scarcely known, and others are mild in character and brief in duration.

Of the emigrants who arrived there in October last, most of them were inhabitants of towns, many of them occupied in sedentary or unhealthy trades, and consequently of feeble and unsound constitutions—numbers landed much afflicted by the scurvy and other diseases of debility, yet, out of about five hundred souls, only two adults have since died, one a worn-out veteran, who had served at Waterloo, another a sick imbecile woman, and two or three infants, all the rest have recovered their strength, enjoy good health, and have increased the population by a number of births.

The writer concludes, by strongly recommending the choice of Auckland to those about to emigrate. " In this genial and equable climate the colonist escapes the seven months Winter of Canada, and its short burning Summer—

the aridity of our African colonies and the withering droughts of Australia. He can assure himself a return for his labour in an abundant harvest, and a profit on his outlay for stock by dairy produce, and the yet unexplored resources of the country are open to his knowledge and perseverance. Here he may acquire a competence without the risk of health, and enjoy a vigor of constitution to which he was a stranger at home, and he can live in a colony where the laws and institutions of Britain, her domestic and social habits are transplanted and have taken root."

